# A second locality for the Namib darkling beetle *Onymacris brainei* (Tenebrionidae, Coleoptera) and first report on its molecular phylogenetic placement

**DOI:** 10.3897/zookeys.687.13660

**Published:** 2017-08-01

**Authors:** Trip Lamb, Eugene Marais, Jason E. Bond

**Affiliations:** 1 Department of Biology, East Carolina University, Greenville, NC 27858, USA; 2 National Museum of Namibia, Windhoek, Namibia; 3 Department of Biological Sciences and Auburn University Museum of Natural History, Auburn University, Auburn, AL 36849, USA

**Keywords:** Adesmiini, Namib Desert, *Onymacris*, Tenebrionidae

## Abstract

*Onymacris brainei* Penrith, 1984 – the most recent species of *Onymacris* to be described – is known only from its type locality in the Namib Desert, adjacent to the Cunene River in northern Namibia. No additional specimens are known to have been collected beyond the type series. Herein, we report on eight specimens discovered at a second site near the original locality. DNA from four beetles was used to determine the phylogenetic placement of *O. brainei* among congeners, based on sequence data from one nuclear (histone III) and two mitochondrial (*cox1*, *cox2*) genes. Maximum likelihood analysis identifies *O. brainei* as a member of the ‘white’ *Onymacris* clade, in which it forms a strongly supported subclade with *O. bicolor* and *O. marginipennis*. More broadly, its phylogenetic placement augments previous molecular results that revealed a sister taxon relationship between the ‘white’ *Onymacris* and a second genus, *Physadesmia*. The paraphyly of *Onymacris* with respect to *Physadesmia* highlights a need for nomenclatural change, but revision should await acquisition of genetic data for the few species outstanding in both genera.

## Introduction

**Figure 1.**
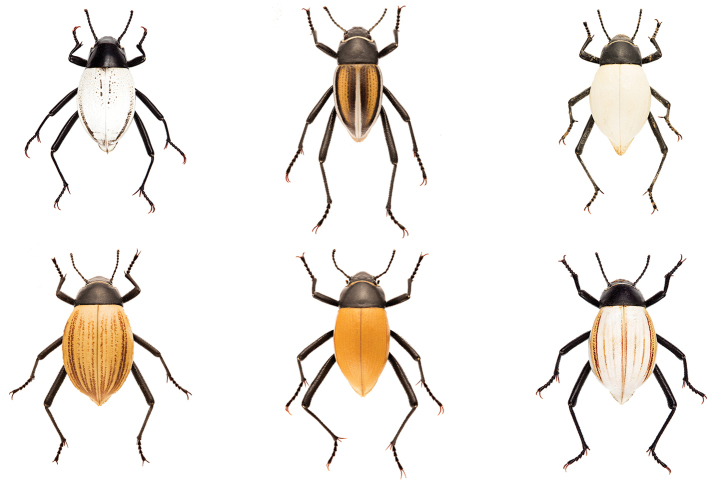
Color variation among members of the ‘white’ *Onymacris* clade, as represented by: (top row, left to right) *Onymacris bicolor*, *O. marginipennis*, *O. candidipennis*, and (bottom row, left to right) *O. langi visseri*, *O. langi cornelii*, and *O. langi meridionalis*.

The darkling beetle genus *Onymacris* is a diverse assemblage of fast-running diurnal species endemic to Africa’s Namib Desert and adjacent southwestern edges of the Kalahari. As substrate specialists, these beetles are restricted to loose sand that characterizes hummocks, dry riverbeds, and dune fields, where they occur in abundance and assume key ecological roles as detritivores (Louw 1983; Hanrahan and Seely 1990). The genus belongs to the flightless tribe Adesmiini, which includes 240+ species and is distributed largely within southwest Africa–a geographic center where adesmiines exhibit their greatest ecological breath and morphological diversity (Koch 1962; Penrith 1979). Regionally, *Onymacris* represents one of the tribe’s more species-rich genera, with 26 currently recognized taxa (14 species and 12 subspecies) that include distinctive ‘white’ species, whose elytral color ranges from pure white to yellow or tan (Fig. 1). White elytral coloration, an unusual characteristic among beetles in general and darkling beetles in particular, is one of many remarkable evolutionary features observed among Namib tenebrionids that are unknown in beetles from other deserts (Hamilton and Seely 1976; Endrödy–Younga 1978; Roberts et al. 1991).

**Figure 2.**
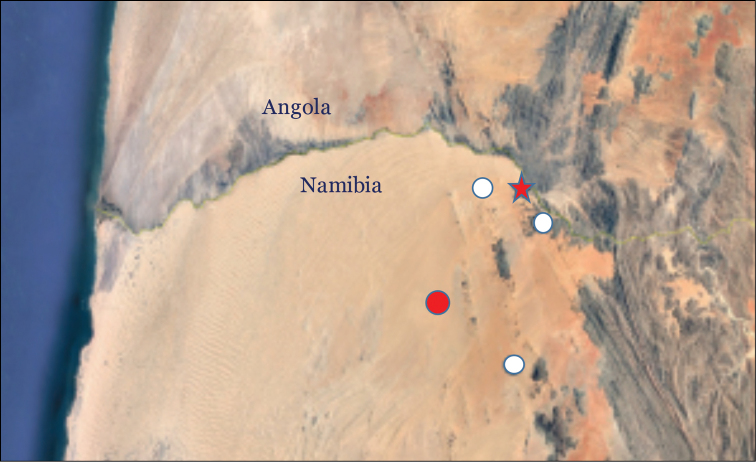
Map illustrating the type locality for *Onymacris brainei* (star), surveyed sites with appropriate habitat (white circles), and the second locality for *O. brainei* (red circle).

**Figure 3.**
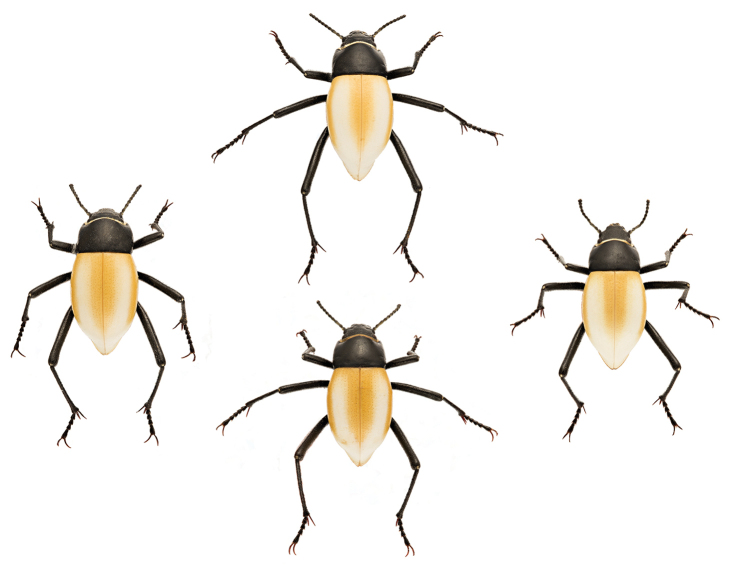
Specimens of *Onymacris brainei* from the second locality, illustrating variation in degree of elytral striping.

‘White’ *Onymacris* are restricted to the northern Namib, where they are patchily distributed, often with limited geographic ranges. *Onymacris brainei*–the most recent member of the genus to be described (Penrith 1984)–represents this case in the extreme: it is known only from the type locality in northern Namibia, just south of the Cunene River along the Angolan border (Fig. 2). Steven Braine collected the first specimens (3 males, 2 females) on 24 February 1983 and brought them to the attention of Mary-Louise Penrith, who at that time was actively revising the southern African Adesmiini (Penrith 1975, 1979, 1984, 1986). Early in the following year (12–15 February 1984), Penrith and Ruth Müller collected 16 additional specimens at the original locality, which provided sufficient material for describing the new species, named in Braine’s honor (Penrith 1984). *Onymacris brainei* is distinguished from other ‘white’ species by the presence of three broad, pale yellow to tan stripes on otherwise white elytra (Fig. 3).

To our knowledge, no other specimens or localities for *O. brainei* have been documented since its description. In 2015, some 30 years after Penrith and Müller’s expedition, we searched the general vicinity of the type locality in an attempt to update the status of *O. brainei*. Herein, we report on eight additional specimens taken from a second site. Importantly, these beetles provided a source of fresh tissue for DNA extraction, gene sequencing, and phylogenetic analysis. Hence we also offer the first report on the molecular phylogenetic placement of *O. brainei* among its congeners.

## Methods

### Field survey for *Onymacris brainei*

Penrith (1984) reported the type locality as “Kunene R. east of dunes at 17.12S, 12.10E,” where beetles were collected “on dune hummocks.” Working from this geographic approximation, we searched a series of appropriate sites (i.e., vegetated hummocks) across the region on 21–22 May 2015. Three of these sites yielded other white *Onymacris* (*O. bicolor*, *O. langi cornelii*), and at a fourth, final site (17°17.87'S; 12°06.20'E), we succeeded in locating *O. brainei* (Fig. 2). Several beetles were observed, of which eight specimens were captured, euthanized (ethanol injection), and carded.

### Molecular phylogenetic analysis

Rear legs from four of the eight beetles were preserved in RNAlater for subsequent DNA isolation using Qiagen’s DNeasy kit. The mitochondrial genes cytochrome oxidase I (*cox1*) and cytochrome oxidase II (*cox2*) and a nuclear gene (histone III, *H3*) were amplified using the primers and PCR conditions listed in Table 1. Amplicons were cleaned using exoSAP-IT (USB Corp.) and sequenced on an Applied Biosystems 3130 capillary sequencer. Sequences were edited and assembled in Sequencher 4.9 (GeneCodes, Ann Arbor, MI) and aligned using ClustalX ver. 2.0 (Larkin et al. 2007), after which sequences were translated to ensure a correct reading frame. Sequences are available through GenBank (Table 2).

**Table 1.** PCR primers and amplification conditions.

Gene	Primer	Annealing	Cycles	Reference
*cox1*	TY-J-1460	50°C	35	Simon et al. (1994)
	TL2-N-3014			
	C1-J-2183	sequencing only		
*cox2*	TL2-J-3037	50°C	35	
	TK-N-3785			
*H3*	Hex AF	61.5°C	45	Odgen and Whiting (2003)
	Hex AR			

DNA sequences for *O. brainei* were combined with sequence data previously generated for *Onymacris* (Table 2) to yield a concatenated dataset–*cox1* (1212 bp), *cox2* (688 bp), and *H3* (317 bp)–representing 18 of the 26 currently recognized species/subspecies. Those taxa unavailable to us for sequencing included *O. candidipennis* and *O. langi langi*, both ‘white’ beetles from Angola, as well as the ‘black’ beetles *O. plana debilis* and *O. paiva conjuncta* (though our dataset contains their nominate subspecies). We also incorporated species sequences representing three additional adesmiine genera: *Physadesmia* (represented by *P. globosa*), shown to be the sister taxon to the white *Onymacris* clade (Lamb and Bond 2013) as well as *Eustolopus octoseriatus* and *Adesmia cribripes*, which served as outgroups.

**Table 2.** GenBank accession numbers for adesmiine sequences used in the ML analysis.

Species	GenBank	GenBank	GenBank
	*cox1*	*cox2*	*H3*
*Onymacris brainei*	MF459686	MF459688	MF459690
*Onymacris brainei*	MF459687	MF459689	—
*O. bicolor*	JX448896	JX448934	JX448972
*O. marginipennis*	JX448907	JX448945	JX448983
*O. langi cornelii*	JX448900	JX448938	JX448976
*O. langi meridionalis*	JX448909	JX448947	JX448985
*O. langi visseri*	JX448921	JX448959	JX448997
*O. boschimana*	JX448897	JX448935	JX448973
*O. multistriata*	JX448912	JX448950	JX448988
*O. hottentota*	JX448901	JX448939	JX448977
*O. plana*	JX448915	JX448953	JX448991
*O. lobicollis*	JX448906	JX448944	JX448982
*O paiva*	JX448913	JX448951	JX448989
*O. rugatipennis*	JX448917	JX448955	JX448993
*O. laeviceps*	JX448904	JX448942	JX448980
*O. u. unguicularis*	JX448919	JX448957	JX448995
*O. u. schulzeae*	JX448920	JX448958	JX448996
*Physadesmia globosa*	JX448887	JX448925	JX448963
*Eustolopus octoseriatus*	JX448886	JX448924	JX448962
*Adesmia cribripes*	JX448889	JX448927	JX448965

We used maximum likelihood (ML) to analyze the concatenated gene dataset. The ML analysis, executed in RAxML ver. 7.2.8 (Stamatakis 2006), comprised 1,000 random sequence addition replicates (RAS) using the commands “-# 1000” and “–m GTRGAMMA.” Bootstrap support values were calculated using the same search parameters with 1,000 replicates, and bootstrap results were applied to the best tree recovered in the RAS search.

## Results

### New locality for *Onymacris brainei*

The second locality for *Onymacris brainei* was discovered on 22 May 2015. Based on the general geographic information provided in Penrith (1984), this new site is estimated to lie ~ 15–20 km SSW of the type locality (Fig. 2). The second site closely resembles the original locality’s physical and ecological description, characterized by vegetated dune hummocks on which nara (*Acanthosicyos horridus*), an iconic Namib endemic, is the prevalent floristic component. Beetles were observed under and, in some cases, on hummock vegetation.

### Elytral color variation

As noted, *Onymacris brainei* is diagnosed by the presence of three broad yellow to tan stripes on white elytra. Specifically, this patterning involves a prominent dorsal stripe that is bisected by the elytral suture and flanked by a slightly narrower lateral stripe on either side. All three stripes bear diffuse edges that coalesce anteriorly near the pronotum, taper posteriorly, and terminate at (or just before) the apical declivity. White elytral coloration is not due to any pigment product but rather a function of reflectivity involving microscopic “bubbles” within the cuticle (Kühnelt 1957). Thus, the stripes represent pigment expression within an otherwise colorless elytral matrix. Penrith (1984) noted that both stripe width and degree of pigment suffusion between stripes varied considerably across the type series. Our eight specimens of *O. brainei* exhibit comparable levels of dorsal color variation (Fig. 3).

### Genetic variation

DNA sequences were invariant for the nuclear gene *H3* but did exhibit variation for both mitochondrial genes (two haplotypes for each gene); mean sequence divergence for the *cox1* and *cox2* was 1.49 % and 0.05%, respectively.

### Molecular phylogenetic placement of *Onymacris brainei*

**Figure 4.**
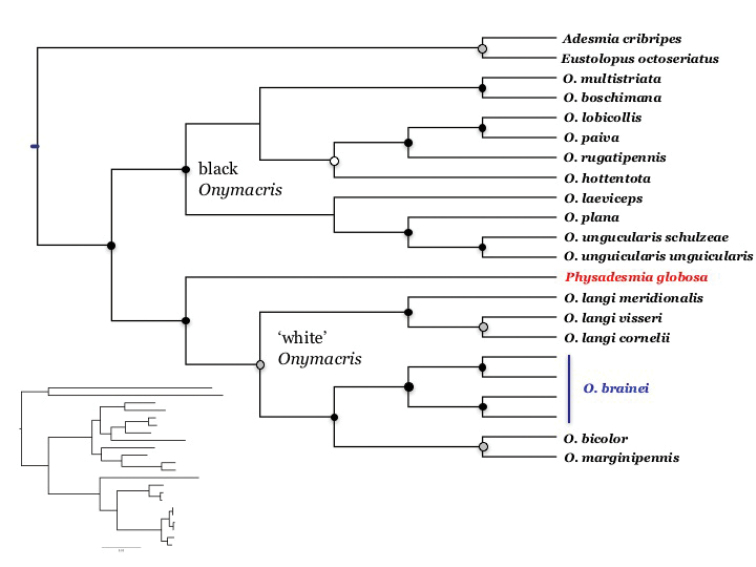
ML consensus topology of *Onymacris*, with bootstrap support indicated by black (> 95%), gray (> 90%), and white (> 70%) nodes. Inset at lower left is a ML tree showing branch lengths.

ML analysis of the concatenated dataset identified *O. brainei* as sister to *O. marginipennis* + *O. bicolor* in a highly supported clade (BS = 100%) that is sister to a second ‘white’ clade comprising the three subspecies of *O. langi* represented in our dataset (Fig. 4). Overall, the ML topology is essentially identical to ML and Bayesian phylogenies previously derived from a larger multilocus dataset (Lamb and Bond 2013), which not only identified two distinct, well supported clades – one containing all ‘white’ species, the other, exclusively black species – but also revealed that *Onymacris* is paraphyletic. All three phylogenies [i.e., this report; Lamb and Bond (2013)] depict *Physadesmia globosa* as the sister taxon to the ‘white” *Onymacris* lineage in a highly supported clade (herein, BS = 99%).

## Discussion

In her paper originally describing *Onymacris brainei*, Penrith (1984) also reported the first cladistic analysis for the genus *Onymacris*, based on 23 morphological characters. To her credit, she examined several additional characters but rejected them “owing to suspected parallelism” or because “the direction of development could not be ascertained.” Her analysis recovered two major clades: an all-black clade comprising six species (*O. boschimana*, *O. laeviceps*, *O. lobicollis*, *O. multistriata*, *O. paiva*, *O. rugatipennis*), and a second clade composed of three additional black species (*O. hottentota*, *O. plana*, *O. unguicularis*) and the ‘white’ species group. Regarding relationships within Penrith’s ‘white’ group, *O. brainei* was placed with *O. bicolor* and *O. marginipennis*, united by the loss of pseudopleural crests along the elytral margins. Furthermore, Penrith’s cladogram depicts *O. brainei* and *O. marginipennis* as sister species on the basis of one synapomorphy–the epistome bearing a deep median emargination.

Our ML phylogeny corroborates *bicolor*-*brainei-marginipennis* monophyly but differs by depicting *O. bicolor* and *O. marginipennis* as sister species. To this end, we note a preliminary aspect of the molecular results–our somewhat limited geographic representation for *O. bicolor* and *O. marginipennis*. Relative to the other ‘white’ taxa, both these species have extended ranges and were recognized historically as being polytypic (Péringuey 1885; Koch 1952). Indeed, *O. bicolor* was for some time treated as two separate species (Koch 1952; Penrith 1975). Thus, while the precise sister status of *O. brainei* remains equivocal (pending further geographic sampling of *O. bicolor* and *O. marginipennis*, particularly Angolan populations), the strongly-supported monophyly of *O. bicolor* + *O. brainei* + *O. marginipennis* is unlikely to change.

The molecular phylogenetic placement of *O. brainei* with other ‘white’ *Onymacris* not only offers incremental support for the ‘white’ clade but, more broadly, augments a diphyletic *Onymacris* relative to *Physadesmia* (Lamb and Bond 2013). Penrith (1979) described the genus *Physadesmia* for three species [*Physadesmia globosa* (Haag), *P. bullata* (Péringuey), and *P. aculeata* (Péringuey)] formerly in *Physosterna*. (Of note, *Physosterna* was subsequently reduced to a subgenus of *Adesmia* (Penrith 1986)). She also observed that “*Physadesmia* and *Onymacris* are evidently very closely related, being separated only by the hypertrophy of the spurs and claws and the shortening of the tarsi in *Onymacris*.” Support for her observation was provided in the first cladistic analysis of adesmiine genera, which recovered a clade comprising *Onymacris*, *Physadesmia*, and a third genus, *Eustolopus* (Penrith 1986). A refined phylogenetic view of *Onymacris*-*Physadesmia*, revealed herein and earlier (Lamb and Bond 2013), identifies a need for nomenclatural changes that will reflect the new found relationship between white *Onymacris* and *Physadesmia*. However, molecular genetic data are still missing for key taxa: two white *Onymacris* (*O. langi langi* and *O. candidipennis*, the latter being the type species of the genus) as well as the remaining two species of *Physadesmia* (*P. bullata* and *P. aculeata*). Though recognizing the necessity for taxonomic change (i.e., either subsuming *Physadesmia* or assigning the black species of *Onymacris* to a new genus), we consider this move to be premature at present and refrain from such effort until relationships for the remaining few species of *Onymacris* and *Physadesmia* have been thoroughly explored.

“Rediscovery” is a beguiling catchword, conveying equal parts accomplishment and optimism upon finding species thought to be rare or possibly extinct. We were indeed relieved to locate new specimens of *O. brainei*–a species gone unreported for 33 years. However, a claim of rediscovery might be overstated: the hiatus is attributable in large degree to the northern Namib’s remote setting and limited accessibility. A more telling discovery may be the genetic divergence (1.49%, *cox1*) observed among individuals at the new locality, which could possibly indicate a historically larger geographic distribution. It is worth noting that *O. candidipennis*, once thought to be restricted to the Namib’s northern terminus in Angola, has been reported from Namibia at the Cunene River, near the type locality for *O. brainei* (Penrith 1984). Moreover, *O. bicolor* and *O. marginipennis*, the two species most closely related to *O. brainei*, occur on both sides of the Cunene. Thus, future assessment on the status of *O. brainei* (regarding genetic variation as well as range delimitation) should involve surveys of suitable habitat from the type locality west to the Cunene mouth, in Angola as well as Namibia. Close proximity of both type and new localities to the contiguous Skeleton Coast (Namibia) and Iona (Angola) national parks offers promise that additional populations of *O. brainei* might be discovered within park boundaries, where they would be afforded full protection.
